# Psychological Morbidity Among Doctors and Nurses at an Indian Tertiary Care Hospital During the COVID-19 Pandemic: A Cross-Sectional Study

**DOI:** 10.7759/cureus.110395

**Published:** 2026-06-07

**Authors:** Yusuf Khan, Zeenat Fatima, Zenab Yusuf Tambawala

**Affiliations:** 1 Emergency Department, East Kent Hospital, Kent, GBR; 2 Obstetrics and Gynecology, Kanad Hospital, Al Ain, ARE; 3 Obstetrics, Dubai Hospital, Dubai, ARE

**Keywords:** anxiety, covid-19, covid-19 pandemic, depression, pandemic, primary healthcare workers, psychological morbidity, stress

## Abstract

Background

Healthcare workers (HCWs) faced substantial psychological pressure during the COVID-19 pandemic. Evidence from Indian tertiary care settings during the acute phase of the outbreak remains limited. This study examined the prevalence of anxiety, depression, and stress among doctors and nurses at a New Delhi tertiary care hospital during the early pandemic period.

Methods

An anonymous, cross-sectional online survey was conducted among 100 HCWs (84 doctors, 16 nurses) at Moolchand Hospital, New Delhi. The Depression, Anxiety and Stress Scale-21 (DASS-21) was used to quantify psychological morbidity. Sociodemographic and occupational data, including infection-control training adequacy, trust in personal protective equipment (PPE) supply, and perceived social support, were collected via a structured questionnaire. Descriptive statistics were used to characterise the cohort and the prevalence of psychological symptoms.

Results

The cohort comprised 53 (53.0%) male and 47 (47.0%) female participants; the majority (40, 40.0%) were aged 31-40 years. Anxiety was the most prevalent psychological outcome (65, 65.0%), followed by depression (20, 20.0%) and stress (15, 15.0%). Insomnia was the most frequently reported individual symptom (43, 43.0%). Participants commonly reported inadequate infection control training, limited trust in PPE adequacy, and self-imposed isolation from family members to reduce transmission risk. Fear of infecting family members and perceived stigma were recurrent qualitative themes.

Conclusions

Psychological morbidity, particularly anxiety, was highly prevalent among doctors and nurses at a New Delhi tertiary hospital during the acute phase of the COVID-19 pandemic. Modifiable factors such as infection control training, PPE provision, and social support emerged as important considerations for institutional response. These findings contribute to the evidence base informing mental health preparedness frameworks for future infectious disease outbreaks, particularly in lower-middle-income country settings.

## Introduction

The COVID-19 pandemic, declared a public health emergency of international concern by the World Health Organization on 30 January 2020 and subsequently a pandemic on 11 March 2020, placed unprecedented strain on healthcare systems worldwide [1]. Healthcare workers (HCWs) on the front line of the response were exposed to elevated occupational, interpersonal, and psychological stressors, including fear of infection, concern for family members, prolonged use of personal protective equipment (PPE), heavy workload, and social stigmatisation [2,3].

Psychological sequelae in HCWs are well-documented following prior epidemics. During the 2003 severe acute respiratory syndrome (SARS) outbreak, studies reported substantial rates of anxiety, insomnia, and burnout among frontline staff [4]. The scale and duration of COVID-19 intensified these pressures considerably. Risk factors identified across earlier outbreaks, including inadequate PPE, heavy workload, fear of infection, and social stigmatisation, were all prominently implicated in the pandemic response [3,5].

Despite a growing international evidence base, data from Indian tertiary care institutions during the acute phase of the pandemic remained limited. This study therefore sought to characterise the prevalence of anxiety, depression, and stress, and to describe associated contextual factors, among doctors and nurses at a major New Delhi hospital during the early pandemic period.

Four years on from the acute phase of the COVID-19 pandemic, systematic reviews and meta-analyses have confirmed that anxiety, depressive symptoms, and burnout persisted well beyond the acute crisis period in many HCW cohorts [6,7]. The mental health burden of the workforce has since been recognised as a major determinant of healthcare capacity, contributing to elevated attrition, reduced clinical performance, and increased risk of medical error [8]. Retrospective analysis of early-pandemic cohorts, therefore, offers an opportunity to identify the modifiable contextual factors that drove this burden and to inform institutional preparedness for future health emergencies, particularly in lower-middle-income country (LMIC) contexts. While meta-analytic estimates provide pooled prevalence figures, they obscure institution-specific contextual drivers, including PPE adequacy, training sufficiency, and social support disruption, that are critical for actionable institutional preparedness. Single-site studies from LMIC tertiary settings therefore retain distinct value for local policy translation.

This study pursued two primary objectives. First, to estimate the prevalence of anxiety, depression, and stress among doctors and nurses at a tertiary Indian hospital during the acute phase of the COVID-19 pandemic. Second, to describe sociodemographic and occupational characteristics relevant to psychological morbidity in this population, with a view to informing institutional mental health preparedness for future infectious disease emergencies.

## Materials and methods

Study design and setting

An anonymous, cross-sectional online survey was conducted at Moolchand Hospital, a tertiary care institution in New Delhi, India, during the acute phase of the COVID-19 pandemic. The survey was administered using a secure Google Forms (Google, Mountain View, CA) link, distributed through internal hospital communication channels. The study protocol was reviewed and approved by the Institutional Review Board and Ethical Committee of Moolchand Hospital and adhered to guidelines from the Indian Council of Medical Research (ICMR) concerning the use of personal information in medical research. Informed consent was obtained from all participants prior to enrolment.

Participants

Eligible participants were licensed doctors and nurses actively practising at Moolchand Hospital during the study period from April 2020 to March 2021, who voluntarily consented to participate. Individuals who were not registered medical professionals, who did not complete the full assessment, or who were medical students without clinical responsibilities were excluded. A target sample of 100 participants was set based on operational feasibility and prevalence estimates of psychological morbidity reported in prior pandemic studies [4,5]. Of the 200 healthcare professionals approached, 157 responded to the survey; 57 were subsequently excluded for incomplete questionnaire completion, yielding a final analytic sample of 100 participants.

Measures

Psychological outcomes were assessed using the Depression, Anxiety and Stress Scale-21 (DASS-21) [9], a validated 21-item self-report instrument quantifying the severity of depressive, anxious, and stress symptoms over the preceding week. Psychological morbidity was classified using established DASS-21 scoring thresholds, in which subscale raw scores are multiplied by two and categorised as follows: depression - normal (0-9), mild (10-13), moderate (14-20), severe (21-27), and extremely severe (28+); anxiety - normal (0-7), mild (8-9), moderate (10-14), severe (15-19), and extremely severe (20+); and stress - normal (0-14), mild (15-18), moderate (19-25), severe (26-33), and extremely severe (34+). As per Lovibond and Lovibond, Cronbach’s α coefficients exceeded 0.80 for all subscales in this cohort, confirming adequate internal consistency [9]. Sociodemographic variables (age, sex, marital status, and urban/rural residence), occupational variables (professional role), and self-reported indicators relevant to the pandemic response (past history of disease or infection, adequacy of infection control training, trust in PPE supply, social support, and self-imposed family isolation) were captured via a structured questionnaire. Open-ended survey items captured contextual experiences, including fear of infection and perceived stigma. Responses were reviewed descriptively; recurring themes are reported as illustrative observations and do not constitute findings from a formal qualitative analysis framework.

The original study protocol also included the Impact of Event Scale-Revised (IES-R). These data are not reported in the present manuscript as data completeness was insufficient for reliable analysis. Accordingly, findings are limited to DASS-21-derived outcomes and do not address post-traumatic stress symptomatology.

Statistical analysis

Data were analysed using IBM SPSS Statistics version 26 (IBM Corp., Armonk, NY). Categorical variables are reported as frequencies and proportions; continuous variables are reported as means with standard deviations (SD) or medians with interquartile ranges, as appropriate. Given the descriptive scope of the study, no formal comparative inferential analyses between subgroups were undertaken.

## Results

The cohort comprised 53 (53.0%) male and 47 (47.0%) female participants. The majority were aged 31-40 years (40, 40.0%), followed by 41-50 years (38, 38.0%), 51-60 years (13, 13.0%), and 18-30 years (9, 9.0%). By professional role, 84 (84.0%) were doctors and 16 (16.0%) were nurses. Most participants were married (89, 89.0%) and held an urban registered place certificate (79, 79.0%). Full demographic characteristics are presented in Table 1.

**Table 1:** Demographic and baseline characteristics of the study cohort (N = 100). Data are presented as n (%).

Characteristic	N (%)
Sex	
Male	53 (53.0)
Female	47 (47.0)
Age group (years)	
18-30	9 (9.0)
31-40	40 (40.0)
41-50	38 (38.0)
51-60	13 (13.0)
Occupation	
Doctor	84 (84.0)
Nurse	16 (16.0)
Marital status	
Married	89 (89.0)
Unmarried	11 (11.0)
Registered residence	
Urban	79 (79.0)
Rural	21 (21.0)

Across the cohort, anxiety was the most prevalent psychological outcome (65, 65.0%), followed by depression (20, 20.0%) and stress (15, 15.0%). Insomnia was the most frequently reported individual symptom (43, 43.0%). The prevalence of psychological symptoms by category is presented in Table 2.

**Table 2:** Prevalence of self-reported psychological symptoms in the study cohort (N = 100). Data are presented as n (%). Symptoms were self-reported and are not mutually exclusive.

Psychological symptom	n (%)
Anxiety	65 (65.0)
Insomnia	43 (43.0)
Depression	20 (20.0)
Stress	15 (15.0)

A substantial proportion of participants reported concerns regarding the adequacy of infection control training and PPE provision during the study period, with more than half indicating limited trust in PPE supply. Participants commonly described self-imposed isolation from family members to reduce perceived transmission risk, and recurrent qualitative themes included fear of infecting loved ones, disruption of family support structures, and perceived social stigmatisation associated with frontline work.

## Discussion

This study documented a high prevalence of psychological morbidity among doctors and nurses at a tertiary Indian hospital during the acute phase of the COVID-19 pandemic. Anxiety was the most prevalent outcome (65, 65.0%), exceeding levels reported in a large Chinese multicentre study (46%) [6] and among Turkish physicians (51.6%) [7]; the reasons for this difference are unclear but may relate to contextual factors, including resource constraints, PPE availability, and training support, as suggested by Indian-context studies [10,11]. Subsequent meta-analyses encompassing data from 2020 to 2023 have confirmed that pooled anxiety prevalence among HCWs globally ranged broadly in the 40-55% range [12-14], situating our findings at the upper end of the international range and underscoring the particular vulnerability of HCWs in LMIC settings. Direct comparison across these studies is limited by differences in sample composition, study timing relative to pandemic waves, healthcare system structure, and instrument cut-off thresholds applied.

Self-reported inadequate infection control training and limited trust in PPE adequacy emerged as the salient contextual concerns in this cohort. These observations are consistent with evidence from other settings linking perceived occupational risk and protective equipment adequacy to psychological distress during the pandemic [8,15]. Pre-existing medical comorbidities, female sex, and disrupted social support were additional factors qualitatively associated with higher psychological symptom burden in this cohort, consistent with prior pandemic literature [7,8,15].

Burnout syndrome, formally recognised by the World Health Organization as an occupational phenomenon under the International Classification of Diseases, 11th Revision (ICD-11) [16], represents a salient concern in HCW populations exposed to sustained pandemic-era working conditions. Longitudinal evidence published since the acute phase has confirmed that burnout trajectories initiated during the COVID-19 crisis persisted into the post-pandemic period for a significant proportion of affected HCWs, contributing to elevated attrition and workforce shortages globally [17,18].

The role of social support deserves emphasis. HCWs in this cohort frequently described voluntary isolation from family members to reduce transmission risk, which may have removed a primary source of emotional resilience and compounded anxiety and depressive symptoms [19]. Targeted psychosocial interventions, including peer-support programmes, structured debriefing, and accessible mental health services, have been proposed as important components of institutional pandemic-response frameworks, though their efficacy requires evaluation in controlled studies. Evidence from post-pandemic evaluations suggests that institutions implementing such programmes during the acute phase reported meaningfully lower rates of long-term psychological morbidity and improved workforce retention [20].

Viewed through the lens of pandemic preparedness, the findings of this study suggest several considerations worthy of further investigation in institutional planning contexts. Infection control training and PPE provision may warrant consideration not only as clinical necessities but as potential contributors to frontline psychological well-being. Social support structures, including family communication policies and flexible rostering, represent plausible components of emergency operational protocols. Similarly, accessible institutional mental health resources throughout and beyond the acute crisis phase may help mitigate the documented persistence of psychological morbidity in HCW cohorts. These observations are hypothesis-generating; their applicability to future pandemic responses, endemic respiratory illness, and other large-scale health emergencies in LMIC healthcare systems requires validation in larger, more representative studies.

Limitations

This study has several important limitations. The sample size was modest (N = 100) and drawn from a single tertiary institution in New Delhi, limiting external generalisability. Data were collected via an anonymous online questionnaire during a strict lockdown period, introducing potential selection bias. The cross-sectional design precludes causal inference, and the absence of longitudinal follow-up prevents characterisation of longer-term psychological trajectories. The cohort was imbalanced by professional role (84 doctors and 16 nurses), which precluded meaningful formal subgroup comparison between these two groups and limits inferences beyond descriptive prevalence. Contextual variables, including infection control training adequacy, PPE trust, and social support, were captured through self-report and are therefore subject to recall and social desirability bias. The study predates widespread vaccination and the emergence of variant strains, and findings may not fully capture the psychological burden across later pandemic waves. The absence of pre-pandemic psychological baseline data for this cohort represents an important limitation. Observed rates of anxiety, depression, and stress cannot be attributed exclusively to pandemic-related exposures; pre-existing vulnerability and occupational stressors may have contributed to the observed burden.

## Conclusions

Psychological morbidity, particularly anxiety, was highly prevalent among doctors and nurses at a single tertiary care institution in New Delhi during the acute phase of the COVID-19 pandemic. Modifiable contextual factors, including inadequate infection control training, limited trust in PPE supply, and disrupted social support, were commonly reported and represent plausible targets for institutional intervention. These findings are hypothesis-generating and should be interpreted within the constraints of a single-site, cross-sectional, descriptive study with a modest sample; larger multisite studies are required before generalisable policy recommendations can be made. Nevertheless, the modifiable contextual factors identified, including PPE adequacy, infection control training, and social support structures, are applicable to future infectious disease preparedness and endemic emergency planning in LMIC healthcare systems, independent of the specific pathogen involved. Policymakers and hospital administrators are encouraged to consider evidence-based psychological interventions, structured training programmes, and inclusive institutional support systems as components of pandemic preparedness frameworks, subject to validation in larger and more representative cohorts. Longer-term longitudinal studies are warranted to assess the durability of pandemic-related psychological effects and the efficacy of targeted institutional interventions.
